# Phonetic detail in German syllable pronunciation: influences of prosody and grammar

**DOI:** 10.3389/fpsyg.2014.00500

**Published:** 2014-05-27

**Authors:** Barbara Samlowski, Bernd Möbius, Petra Wagner

**Affiliations:** ^1^Work Group Phonetics/Phonology, Faculty of Linguistics and Literary Studies, Bielefeld UniversityBielefeld, Germany; ^2^Department of Computational Linguistics and Phonetics, Saarland UniversitySaarbrücken, Germany

**Keywords:** prominence, duration, stress, syntactic boundaries, lexical class, lemma frequency

## Abstract

This study presents two experiments designed to disentangle various influences on syllable pronunciation. Target syllables were embedded in carrier sentences, read aloud by native German participants, and analyzed in terms of syllable and vowel duration, acoustic prominence, and spectral similarity. Both experiments revealed a complex interaction of different factors, as participants attempted to disambiguate semantically and syntactically ambiguous structures while at the same time distinguishing between important and unimportant information. The first experiment examined German verb prefixes that formed prosodic minimal pairs. Carrier sentences were formulated so as to systematically vary word stress, sentence focus, and the type of syntactic boundary following the prefix. We found clear effects of word stress on duration, prominence, and spectral similarity as well as a small influence of sentence focus on prominence levels of lexically stressed prefixes. While sentence boundaries were marked by particularly high prominence and duration values, hardly any effect was shown for word boundaries. The second experiment compared German function words which were segmentally identical but appeared in different grammatical roles. Here, definite articles were found to be shorter than relative pronouns and still shorter than demonstrative pronouns. As definite articles are also much more common than the other two lexical classes, effects of lemma frequency might also have played a role.

## Introduction

Syllables can vary strongly in the way they are pronounced, even when in canonical pronunciation they are segmentally identical. One important source of variation is prominence, i.e., the degree of emphasis which is placed on syllables and with which they are perceived. Such emphasis may be realized by means of higher duration and intensity values, overall larger articulatory effort, as well as the presence and shape of pitch accents (Wagner, 2002). Among other things, prominence differences are used to distinguish between lexically stressed and unstressed syllables. Duration seems to be a main correlate of word stress in German, but differences were also found for formant values, fundamental frequency, and various voice quality parameters (e.g., Kohler, 1987; Claßen et al., 1998; Kleber and Klipphahn, 2006; Schneider and Möbius, 2007; Lintfert, 2010). Studies specifically investigating word stress in focused and unfocused sentence positions have confirmed duration as a strong signal of word stress which operates independently of sentence accent (Dogil and Williams, 1999 for German; Okobi, 2006 and Cho and Keating, 2009 for English; Sluijter and van Heuven, 1996 for Dutch). However, for English, Plag et al. (2011) found no effect at all of word stress on duration, while Campbell and Beckman (1997) discovered stress-related duration differences only in one of the two unaccented contexts examined. For English and Dutch, spectral tilt, i.e., the intensity in higher compared to lower frequency bands, appeared to be another robust correlate of word stress in accented as well as unaccented contexts (Sluijter and van Heuven, 1996; Okobi, 2006; Plag et al., 2011). Although Dogil and Williams (1999) found no significant differences between accented and unaccented words in German in terms of fundamental frequency, intensity, or duration, studies for other languages showed stress-related differences in fundamental frequency and intensity to be strongly reduced when target words were not accented (Sluijter and van Heuven, 1996; Plag et al., 2011). Apart from signaling sentence focus, prominence differences are also used to distinguish important from unimportant information on the level of lexical class. In German speech synthesis, lexical class has been used as an important indicator for predicting prominence levels (Widera et al., 1997; Windmann et al., 2011). Frequency and predictability effects have an influence on word pronunciation as well. There is evidence for English that words tend to be spoken at a faster rate if they are frequent or easily predictable from their context (Bell et al., 2003; Aylett and Turk, 2004; Baker and Bradlow, 2009). Although effects of word frequency and lexical class are often confounded, both factors were found to play an important role (Jurafsky et al., 2000; Pluymaekers et al., 2005; Bell et al., 2009). The present study consists of two controlled production experiments. The first experiment aims to disentangle influences of lexical stress, sentence accent, and syntactic boundaries, while the second experiment analyzes effects of lexical class and word frequency.

## Methods

### Participants

Thirty participants took part in the two experiments (15 men, 15 women, ages ranging between 19 and 47). All were native speakers of German. They were paid for their participation in the study.

### Material

#### Experiment 1: stress, accent, and syntactic boundaries

Certain German verbs can differ in meaning depending on whether their lexical stress falls on the prefix or the verb stem. For example, the word [ɂʊn.tɐ.ʃtε.lən] ([unter]_prefix_−[[stell]_stem_−[en]_ending_]_verb_), literally “to underput”) means “to store / take shelter” when stressed on the prefix, but “to insinuate” when stressed on the stem. This ambiguity is not visible in all inflections, however. In most finite forms, lexically stressed prefixes are separated from the verb and placed at the end of the clause. As the verb prefixes used for this experiment are segmentally identical to prepositions or conjunctions, we were able to use them to analyze effects of syntactic boundaries as well. We examined the effects of word and sentence stress as well as word and sentence boundaries on the production of the four German verb prefixes “um” ([ɂʊm] – “around”), “unter” (['ɂʊn.tɐ] – “under”), “über” (['ɂyː.bɐ] – “over”), and “durch” ([dʊʁç] – “through”) in a reading task (see also Samlowski et al., 2012). The phonetic transcriptions given here are canonical. The glottal stop preceding onset vowels may be omitted or realized through vowel glottalization, and the [ʁ] in “durch” is commonly rendered as [ɐ].

Target items consisted of the prefixes combined with two different verb stems each. Each of the eight resulting verbs was placed in seven different carrier sentences. In sentences 1–4, word and sentence stress were varied, while sentences 5–7 compared different types of syntactic boundaries (see Table 1). As participants needed to be able to infer the correct stress pattern from the sentence context, a different set of carrier sentences was created for each verb. Sentence stress differences were not elicited in a uniform manner, either. While sentences belonging to the categories “w+s+” and “w−s+” were formulated so as to imply a broad focus, sentences in categories “w+s−” and “w−s−” contained elements designed to attract a contrasting focus and thereby move the sentence stress away from the main verb. Among the strategies used for this were the inclusion of two contrasting objects, topic fronting, and the addition of an emphasized modifier. For the sake of brevity in this paper we refer to the first four sentence categories in terms of stressed and unstressed prefixes (“w+” vs. “w−”) in accented and unaccented conditions (“s+” vs. “s−”). Nonetheless, it is important to note that the categories do not reflect the actual stress patterns used by the participants. Instead they describe potential differences in word and sentence stress due to different word meanings and the presence or absence of an additional motivation for deaccentuating the verb. Our aim is to discover the extent to which these conceptual differences are realized in the acoustic production of the target syllables.

**Table 1:** **Sentence categories**.

**Category**	**Canonical word stress**	**Additional semantic contrast**	**Right-hand boundary**
w+s+	**Yes**	**No**	(morpheme)
w+s−	**Yes**	**Yes**	(morpheme)
w−s+	**No**	**No**	(morpheme)
w−s−	**No**	**Yes**	(morpheme)
sb	(undefined)	(no)	**Sentence**
mb	(no)	(no)	**Morpheme**
wb	(undefined)	(no)	**Word**

*Word and sentence stress status and succeeding syntactic boundary for the target items in each of the 7 sentences (manipulated parameters in bold)*.

We deliberately decided against using underline, font style, or a question-answer structure to indicate lexical stress and sentence focus, since we wanted to avoid potentially evoking exaggerated responses by attracting the participants' attention to the intended reading. This meant that the context was not controlled across verbs and only up to a limited degree within each set of sentences. For the first four sentences within one set, the half syllable preceding and following the prefix were kept constant. Sentence 5 (“sb”) used the same preceding half-syllable as the first four. While the prefix in sentence 6 (“mb”) fulfilled the same conditions as in sentence 3 (“w−s+”), its target sentence was formulated so that the preceding and following half-syllables matched those of the identical prepositions or conjunctions in sentence 7 (“wb”).

#### Experiment 2: lexical class and word frequency

While different words are used for German demonstrative pronouns, relative pronouns, and definite articles, depending on gender, number, and case, these words are often segmentally identical across the three lexical classes. Definite articles are much more common than the segmentally identical demonstrative or relative pronouns. According to the DeWaC corpus (Baroni and Kilgarriff, 2006), a 1.5 billion word database of German internet articles which was automatically tagged for lexical classes, the words “der” ([deːɐ]), “die” ([diː]), “das” ([das]), “dem” ([deːm]), and “den” ([deːn]) were used as definite articles 89.8% of the time, while 7.2% of their appearances were classified as relative pronouns, and only 3% were demonstrative pronouns.

To examine whether these differences in frequency of occurrence have an influence on pronunciation, we compared their realizations in different grammatical roles (see also Samlowski et al., 2013). Sentences containing relative and demonstrative pronouns were formulated so as to match definite articles already appearing in one of the other carrier sentences from the two experiments. As each of the lexical classes required different types of surrounding grammatical structure, only the half-syllables preceding and following the target word were held constant across each group of 3 sentences. For each of the investigated words, 3 sentence groups were assembled (see Table 2), resulting in a total of 48 new sentences containing relative and demonstrative pronouns.

**Table 2:** **Target items**.

**Orthographic form**	**Phonetic transcription**	**Gender / number / case**	**No. of stimuli**
der	[deːɐ]	**Masculine** singular nominative ***or***	3 × 3
		**Feminine** singular dative	3 × 3
die	[diː]	Feminine **singular** nominative/accusative ***or***	3 × 3
		Masculine/feminine/neuter **plural** nominative/accusative	3 × 3
das	[das]	Neuter singular nominative/accusative	3 × 3
dem	[deːm]	**Masculine** singular dative ***or***	3 × 3
		**Neuter** singular dative	3 × 3
den	[deːn]	Masculine singular accusative	3 × 3

*Orthographic form, phonetic transcription, grammatical description and number of stimuli used for each word*.

### Procedure

**Figure 1:**
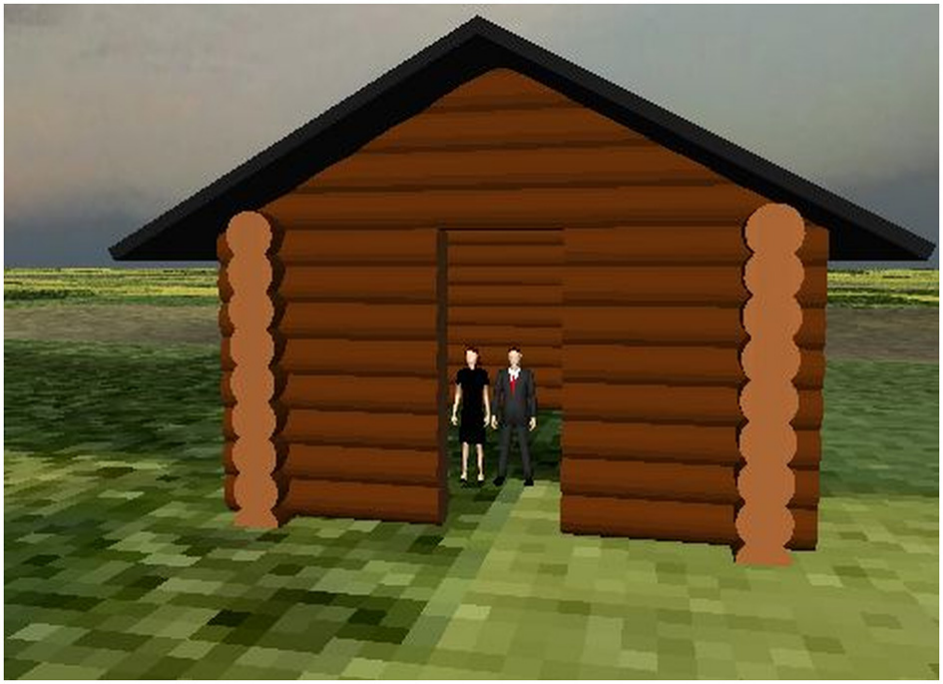
**Example illustration for Experiment 1—“unterstellen” (category “w+s+”)**. Corresponding sentence: “Wir wollten uns ***unterstellen***, weil es so stark regnet.” (English: “We wanted to ***take shelter*** because it is raining so heavily.”)

**Figure 2:**
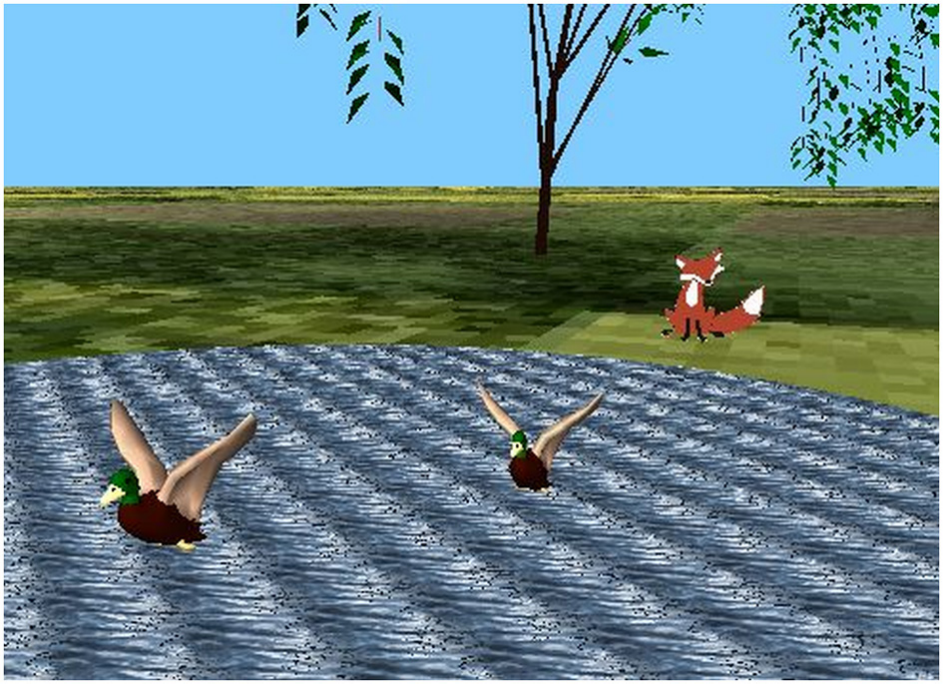
**Example illustration for Experiment 2—“den” (rp)**. Corresponding sentence: “Es war deutlich, dass der Fuchs den See beobachtete, ***den*** Enten als ihre Heimat gewählt hatten.” (English: “It was clear that the fox was watching the lake ***which*** ducks had chosen as their home.”)

Sentences from both experiments were placed in a quasi-random order, which was not varied across participants. Care was taken to avoid repetitions of the same verb and provide a good mixture of sentences from both experiments, allowing them to function as mutual distractors. Acoustic recordings took place in a sound-treated chamber at Bielefeld University. One sentence at a time was presented on a computer screen to the participants, who proceeded through the experiment in a self-paced manner. To further clarify the intended word meaning and improve understanding of the reading content, sentences were illustrated using the text-to-scene conversion program WordsEye (Coyne and Sproat, 2001, see Figures 1 and 2). Participants looked at each sentence and the accompanying picture and then read the sentence out loud. Beforehand, they were instructed to repeat any sentences in which they made a mistake or slip of the tongue. These sentences as well as sentences where participants hesitated noticeably while reading were omitted from analysis. Target items were also discarded if they or their immediate context was impaired through speech errors, noise, or unexpected vowel elision.

The remaining recordings were analyzed in terms of syllable and vowel duration, acoustic prominence, and spectral similarity. For the duration and prominence analysis, syllable and vowel boundaries of the target items as well as the preceding and following syllables were manually annotated with Praat (Boersma, 2001). Acoustic prominence was investigated by means of an automatic prominence tagger which analyzed annotated syllable nuclei in terms of pitch movement, duration, intensity, and spectral emphasis. Values for the last three parameters were normalized across all investigated syllables in the utterance using *z*-scores and the individual factors were weighted so as to model perceptual ratings of German prominence (Tamburini and Wagner, 2007). In the present study, only the syllables immediately preceding or following the target items were used as context for the tagger. If the vowel of a context syllable tended to be elided, the preceding/following syllable nucleus was taken as context syllable instead. We also compared pairs of segmentally identical syllables produced by the same speaker in terms of spectral similarity, using a method developed by Wade and Möbius (2007) and Lewandowski (2011). Amplitude envelopes were computed for 4 frequency bands (equally spaced on a logarithmic scale ranging from 80 to 7800 Hz), using a sampling rate of 500 Hz. The spectral similarity of two syllables was calculated by cross-correlating pairs of envelopes for each frequency band, taking the maximum of the cross-correlation as an indicator for the degree of similarity. Although spectral similarity is not a direct measure of vowel quality and degree of coarticulation, it can serve as an indication of how strongly the target items varied in their pronunciation across contexts and categories. Statistical analysis and visualization was performed with R (R Development Core Team, 2010). As residuals from analyses of variances only followed a normal distribution in the case of the duration results of the second experiment, the other investigations were analyzed with Wilcoxon rank sum tests. Significance values were Bonferroni-corrected for multiple comparisons.

## Results

### Experiment 1: stress, accent, and syntactic boundaries

Of the 1680 sentences collected (8 verbs × 7 sentences × 30 participants), 113 were discarded. As two of the prefixes used are bisyllabic, the following analyses are based on a total of 2278 syllables. The results were analyzed in terms of sentence category (“w+s+,” “w+s−,” “w−s+,” “w−s−,” “sb,” “mb,” “wb”) and syllable identity ([ɂʊm], [ɂʊn], [tɐ], [?y:], [bɐ], [dʊʁç]).

#### Duration

**Figure 3:**
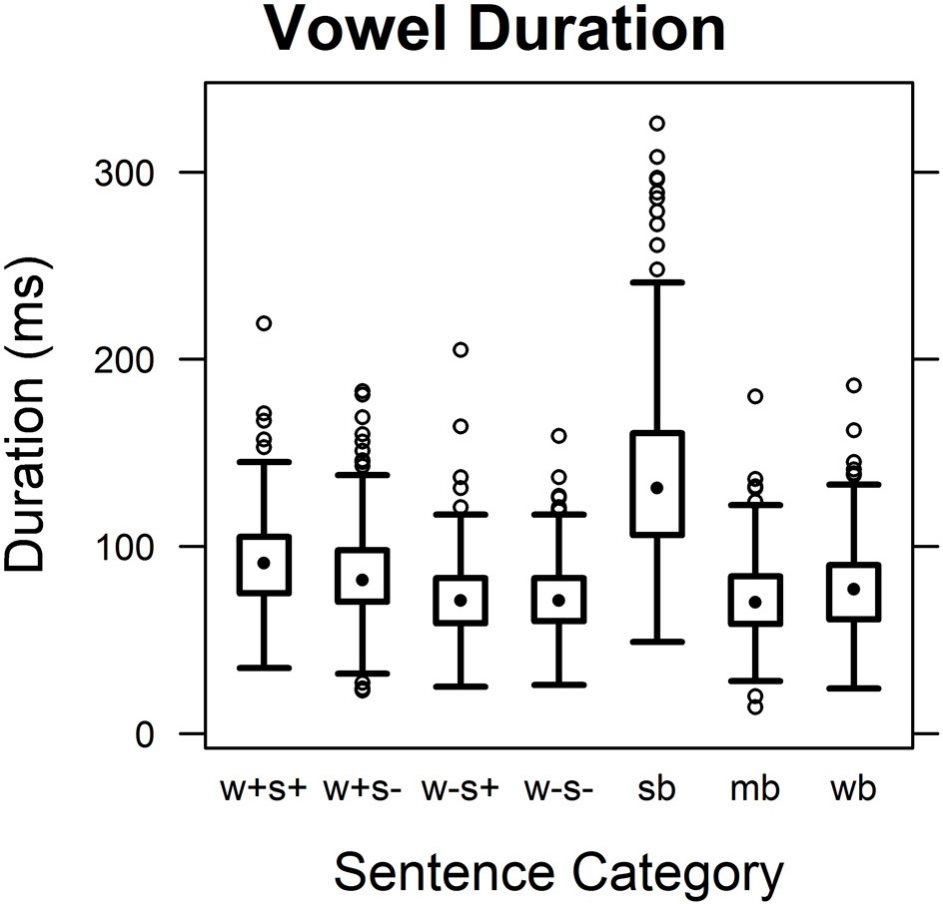
**Vowel duration**. Duration values in across syllables for the seven sentence categories.

Figure 3 gives an overview of vowel duration results for the seven sentence categories examined. Wilcoxon rank sum tests comparing sentence categories across syllables (corrected for 21 comparisons) showed phrase-final prefixes (“sb”) to be significantly longer than those in the other categories (*W* > 87,000, *p* < 0.0001). A small influence of word stress was also observed, with syllables and vowels being longer when appearing in lexically stressed compared to unstressed prefixes (“w+s+” vs. “w−s+,” “w+s−” vs. “w−s−,” *W* > 62,000, *p* < 0.0001). Vowel duration of lexically stressed prefixes was slightly reduced if the verb was not in the focus of the sentence (“w+s+” vs. “w+s−,” *W* = 59,074, *p* < 0.05). Finally, there was a small tendency for prepositions or conjunctions to have slightly longer syllables and vowels than segmentally identical bound prefixes (“wb” vs. “mb,” *W* > 59,000, *p* < 0.05).

Syllable and vowel durations were also analyzed for combinations of syllable identity and sentence category (corrected for 861 comparisons, see Table 3 for mean values). All investigated syllables were significantly longer when they occurred in separated sentence-final prefixes than in other contexts (“sb” vs. others, *W* > 2400, *p* < 0.0001). Differences in vowel duration were significant for all syllables except [ɂʊn]. Here, differences between separated prefixes and bound prefixes in lexically stressed and potentially accented positions (“sb” vs. “w+s+”) failed to reach significance, and comparisons between separated prefixes and segmentally identical function words (“sb” vs. “wb”) were significant on a lower level (*W* = 2386, *p* < 0.01) than the other comparisons (*W* > 2200, *p* < 0.0001). No significant influences were shown for word boundary (“mb” vs. “wb”) or sentence stress (“w+s+” vs. “w+s−,” “w−s+” vs. “w−s−”). Effects of word stress on syllable and vowel duration are summarized in Table 4.

**Table 3:** **Mean duration**.

**Category**	[ɂʊm]	[ɂʊn]	[tɐ]	[?y:]	[bɐ]	[dʊʁÇ]	**All**
w+s+	147.6	142.4	132.1	90.0	138.0	199.1	140.0
	72.2	88.6	88.0	90.0	87.5	114.4	91.7
w+s−	151.4	130.7	125.6	84.8	136.6	194.7	137.0
	77.1	79.4	79.2	84.8	85.5	110.2	85.6
w−s+	143.4	112.6	115.5	59.9	126.3	149.0	117.0
	71.6	66.1	74.7	59.9	75.7	81.2	71.3
w−s−	140.5	112.5	114.5	66.9	127.0	161.9	120.7
	66.5	65.5	73.0	66.9	77.2	88.7	72.9
sb	261.0	185.2	205.5	132.3	207.1	414.2	228.7
	128.6	106.3	137.8	132.3	147.1	188.0	140.1
mb	127.0	116.3	119.5	59.6	121.9	146.9	114.5
	61.7	71.1	76.4	59.6	74.1	83.5	71.2
wb	140.2	127.8	124.5	71.4	127.8	152.8	124.4
	71.9	82.5	76.5	71.4	76.9	84.3	77.3

*Mean duration values in milliseconds for syllables (above) and vowels (below) in the seven sentence categories*.

**Table 4:** **Duration statistics for lexical stress**.

**Category**	[ɂʊm]	[ɂʊn]	[tɐ]	[?y:]	[bɐ]	[dʊʁÇ]
w+s+ vs. w−s+	1868.0 (n.s.)	2485.0^****^	2214.5^*^	2687^****^	2264.5 (n.s.)	2063.5^****^
	1756.5 (n.s)	2423.5^****^	2177.5 (n.s.)	2687^****^	2365.5^**^	2199^****^
w+s− vs. w−s−	1743.0 (n.s.)	2353.5^*^	2150.0 (n.s.)	1921^*^	1670.5 (n.s.)	1925.5 (n.s.)
	1870.5 (n.s.)	2309.5^*^	2116.5 (n.s.)	1921^*^	1674 (n.s.)	1945.4^*^

W values with significance levels for syllables (above) and vowels (below), n.s.: p ≥ 0.05,

* p < 0.05,

** p < 0.01,

**** *p < 0.0001*.

#### Prominence

**Figure 4:**
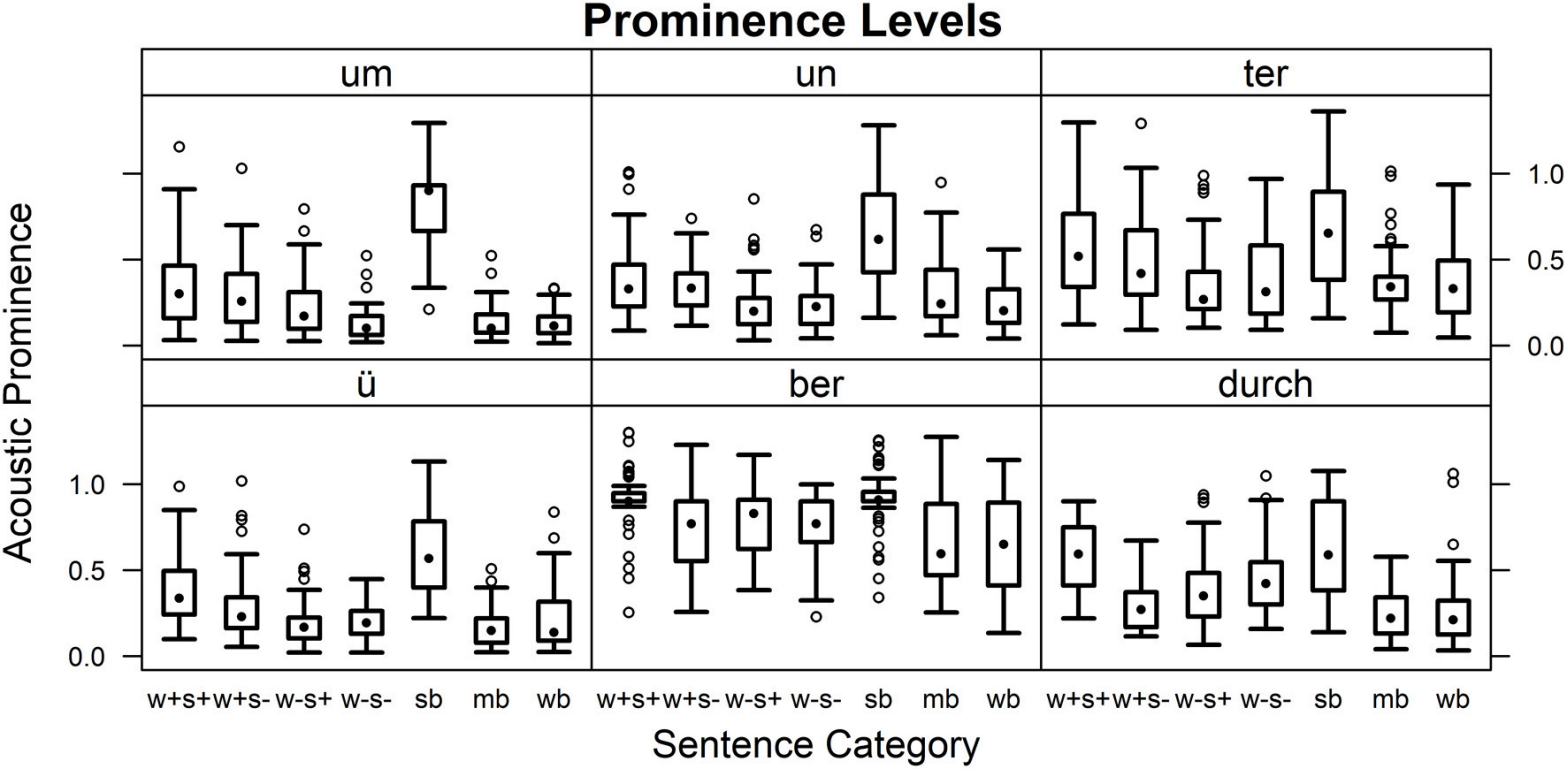
**Prominence values**. Estimates of acoustic prominence for the individual syllables in the seven sentence categories.

Prominence estimates for the individual syllables in the seven sentence categories are shown in Figure 4. Wilcoxon rank sum tests for sentence categories across syllables (corrected for 21 comparisons) showed that lexically stressed prefixes tended to receive significantly higher prominence values than unstressed ones in accented as well as unaccented conditions (“w+s+” vs. “w−s+,” *W* = 72,651, *p* < 0.0001; “w+s−” vs. “w−s−,” *W* = 59,677, *p* < 0.01). Sentence stress differences were significant for lexically stressed prefixes (“w+s+” vs. “w+s−,” *W* = 63,503, *p* < 0.0001). Separated, phrase-final prefixes were particularly high in prominence (“sb” vs. others, *W* > 76,000, *p* < 0.0001), while no effect of word boundary was observed (“mb” vs. “wb”).

In tests for combinations of sentence categories and syllables (corrected for 821 comparisons) differences related to word and sentence stress mostly failed to reach significance. Word stress effects were found for [ɂyː] and [dʊʁç] in accented conditions as well as for [ɂʊm] and [ɂʊn] in unaccented conditions (see Table 5). Effects of sentence stress were only shown in the case of lexically stressed [dʊʁç] (“w+s+” vs. “w+s−,” *W* = 2189, *p* < 0.0001). In separated, phrase-final prefixes, syllables often received significantly higher prominence values than in the other categories (“sb” vs. others, *W* > 1900, *p* < 0.05). Exceptions for this last tendency were found for [tɐ] (“sb” vs. “w+s+,” “sb” vs. “w+s−”), [bɐ] (“sb” vs. “w+s+,” “sb” vs. “w−s+”), and [dʊʁç] (“sb” vs. “w+s+,” “sb” vs. “w−s−”). No significant differences appeared between bound prefixes and corresponding prepositions or conjunctions (“mb” vs. “wb”) or between unstressed prefixes in accented and unaccented conditions (“w−s+” vs. “w−s−”).

**Table 5:** **Prominence statistics for lexical stress**.

**Categories**	[ɂʊm]	[ɂʊn]	[tɐ]	[?y:]	[bɐ]	[dʊʁÇ]
w+s+ vs. w−s+	1922.5 (n.s.)	2171.5 (n.s.)	2174 (n.s.)	2669.5^****^	2085 (n.s.)	1977^**^
w+s− vs. w−s−	2357.5^****^	2307.5^*^	1985 (n.s.)	1664.5 (n.s.)	1240.5 (n.s.)	741 (n.s.)

W values with significant levels for comparisons between lexically stressed and unstressed prefixes, n.s.: p ≥ 0.05,

* p < 0.05,

** p < 0.01,

**** *p < 0.0001*.

#### Spectral similarity

For each target syllable in each sentence category, we calculated the level of similarity between prefixes produced by the same speaker in the two verb contexts. Wilcoxon rank sum tests comparing sentence categories across syllables (corrected for 21 comparisons) showed significant differences in syllable similarity for stressed versus unstressed prefixes in accented conditions (“w+s+” vs. “w−s+,” *W* = 16,183.5, *p* < 0.01, mean values: 0.889 vs. 0.848). Sentence stress differences in stressed prefixes had only a marginally significant effect (“w+s+” vs. “w+s−,” *W* = 14,189.5, *p* = 0.052, mean values: 0.889 vs. 0.864). Separated, phrase-final prefixes (“sb,” mean: 0.897) received significantly higher similarity values (*W* > 16,000, *p* < 0.001) compared to all examined categories except for stressed and potentially accented prefixes (“w+s+”). Effects were most pronounced for the syllables [ɂyː], [dυʁç], and, to a lesser extent, [ɂʊn], although results failed to reach significance when combinations of syllables and sentence categories were investigated (corrected for 821 comparisons).

In an analysis of spectral similarity between sentence categories for syllables produced by the same speaker in the same verb context, comparisons with separated, phrase-final prefixes tended to result in lower values than comparisons between the other sentence categories (“sb” vs. others). This effect was shown to be significant (*W* > 57,000, *p* < 0.0001) in tests for combinations of sentence categories (corrected for 210 comparisons). Lexically stressed prefixes were significantly closer to those in sentence-final prefixes than syllables in unstressed prefixes (“w+s+” and “sb” vs. “w−s+” and “sb,” “w+s−“ and “sb” vs. “w−s−” and “sb,” *W* > 64,000, *p* < 0.001, mean values: 0.794 vs. 0.748 and 0.799 vs. 0.765). Here as well as for the comparisons within sentence categories, effects were most clearly visible for [ɂyː] and [dʊʁç]. An analysis of similarity between sentences in the “sb” category and those in the other categories combined with syllable identity (corrected for 630 comparisons) showed significant differences between stressed and unstressed [ɂyː] in accented conditions (“w+s+” and “sb” vs. “w−s+” and “sb,” mean values: 0.816 vs. 0.709, *W* = 2552, *p* < 0.0001).

#### Discussion

Apart from Dogil and Williams (1999), there have been hardly any studies examining the interaction of word and sentence stress in German. In our paper, we examine the extent to which canonical word stress differences and additional semantic contrasts triggered differences in the word and sentence stress patterns which in turn were visible in the acoustic realization of the target syllables. Based on German language corpus studies as well as evidence from other Germanic languages, we expected lexically stressed syllables to be longer than unstressed syllables in accented as well as unaccented conditions. We also predicted an effect of word and sentence stress on acoustic prominence levels compared to the immediate surroundings. Although spectral parameters have been shown to be affected by stress, we had no clear hypotheses as to how word and sentence stress might influence similarity across and within sentence categories. Our study indeed showed a significant influence of lexical stress on duration values for all investigated prefixes apart from [ɂʊm]. When sentences were given a broad focus, even the lexically unstressed second syllables of the prefixes ['ɂʊn.tɐ] and ['ɂyː.bɐ] were affected. This result may be explained by accentual lengthening of the word carrying sentence stress, as there is evidence that in English and Dutch this effect is stronger to the right of the lexically stressed syllable than to the left (Cambier-Langeveld and Turk, 1999). There was also a tendency for stressed syllables to be higher in prominence and more similar to syllables in sentence-final prefixes than unstressed ones. When no deaccentuation cues were given, lexically stressed syllables were more similar across verb contexts than unstressed syllables. Results for prominence and spectral similarity mostly failed to reach significance in a syllable-by-syllable analysis. One reason for the small size of the word stress effects might be that all investigated syllables except [ɂyː] had lax vowels, since these have been found to have a considerably reduced effect of lexical stress on duration (Mooshammer et al., 1999; Kleber and Klipphahn, 2006). Although there was a slight effect of sentence stress on duration and prominence values of lexically stressed syllables, it almost never reached significance in a syllable-by-syllable analysis. Although the data was not analyzed perceptually, auditory impressions suggest that participants often placed a secondary accent on the target verb in unaccented conditions—perhaps because they wanted to better clarify the intended word meaning or because the given cues were not strong enough. Particularly in the case of the verbs ['dʊʁç.ʃaʊ.ən] (“to look through”) and ['ʊm.faː.ʁən] (“to run over”), effects of final lengthening might also have played a role, as these were sentence-final in the unaccented, but not in the accented conditions. The unusually strong effect of sentence stress on prominence levels for [dʊʁç] may have been due to the fact that ['dʊʁç.ʃaʊ.ən] was one of the few verbs where the potentially contrasting sentence stress in the unaccented condition would actually fall on the syllable used as preceding context by the tagger.

As was to be expected, a large effect of sentence boundary on syllable and vowel duration was observed. All examined syllables, including the first syllables of the prefixes ['ɂʊn.tɐ], and ['ɂyː.bɐ], were considerably lengthened when appearing in sentence-final, separated prefixes. The results confirm findings by Kohler (1983) and Silverman (1990), according to which sentence-final lengthening extends beyond the final syllable. Effects of sentence boundary were also found for prominence and spectral similarity, although not all syllables were affected equally. The interpretation of possible word boundary effects is not straightforward. A longer duration of free words might be expected due to effects of word-final lengthening (e.g., Beckman and Edwards, 1990) or polysyllabic shortening (e.g., Turk and Shattuck-Hufnagel, 2000; White, 2002), as bound prefixes were not followed by a word boundary and therefore appeared in longer words than the corresponding prepositions or conjunctions. Also, bisyllabic items had lexical stress on the first syllable as free words, but not as bound prefixes. On the other hand, there might have been counteracting influences of word frequency and accentual lengthening, as the verbs used were generally less frequent than the matching function words and tended to attract sentence focus. In our study, syllables in bound prefixes tended to be slightly shorter than when they occurred in segmentally identical prepositions or conjunctions, with the first syllable of the bisyllabic ['ɂʊn.tɐ] and ['ɂyː.bɐ] being affected more strongly than the second syllable. No influence was found for prominence and similarity values, and the word boundary effect was not significant in a separate investigation of the individual target syllables.

### Experiment 2: lexical class and word frequency

Of the 2160 items recorded (8 words × 3 contexts × 3 lexical classes × 30 participants), 310 had to be omitted from the analysis. Results are based on the remaining 1850 items, which were investigated with regards to the factors lexical class (“dp,” “rp,” “da”) and word identity (“der masc.,” “der fem.,” “die sg.,” “die pl.,” “das,” “dem masc.,” “dem neut.,” “den”).

#### Duration

In terms of syllable and vowel duration, demonstrative pronouns tended to be slightly longer than segmentally identical definite articles, with relative pronouns usually falling somewhere in between. This trend was especially noticeable for feminine “der” as well as masculine and neuter “dem.” Differences for “den,” masculine “der,” and singular “die” were less pronounced, while hardly any changes were observed for “das” and plural “die” (see Table 6 for mean values). Two-Way ANOVAs were computed to examine the influence of word identity and lexical class on log-transformed syllable and vowel duration values. Significant effects (*p* < 0.0001) were found for word identity [syllable duration: *F*_(7, 1824)_ = 147.8, vowel duration: *F*_(7, 1824)_ = 61.2], lexical class [syllable duration: *F*_(2, 1824)_ = 123.2, vowel duration: *F*_(2, 1824)_ = 109.3], and their interaction [syllable duration: *F*_(14, 1824)_ = 8.6, vowel duration: *F*_(14, 1824)_ = 11.0]. Tukey's HSD tests were used to further investigate the data. In terms of syllable as well as vowel duration, significant differences (*p* < 0.001) were found between masculine and neuter “dem” and between masculine and feminine “der,” but not between singular and plural “die.” Significance levels for the interaction between lexical class and word identity are given in Table 7.

**Table 6:** **Mean duration**.

**Lexical class**	**der (masc.)**	**der (fem.)**	**die (sg.)**	**die (pl.)**	**das**	**dem (masc.)**	**dem (neut.)**	**den**	**All**
dp	150.0	193.4	124.7	126.7	186.7	223.5	185.5	200.8	175.0
	96.9	139.4	79.4	71.6	72.7	84.2	85.6	82.7	89.8
rp	124.6	144.5	126.8	117.1	186.8	212.9	172.2	189.1	158.0
	78.3	101.8	68.0	64.4	60.5	76.7	76.5	73.9	74.8
da	118.2	104.3	100.0	112.3	182.0	161.9	141.9	150.7	133.6
	74.7	81.9	65.5	66.7	71.6	45.8	61.6	58.6	65.8

*Mean duration values of demonstrative pronouns (dp), relative pronouns (rp) and definite articles (da) in milliseconds for syllables (above) and vowels (below)*.

**Table 7:** **Interaction of lexical class and word identity**.

**Lexical class**	**der (masc.)**	**der (fem.)**	**die (sg.)**	**die (pl.)**	**das**	**dem (masc.)**	**dem (neut.)**	**den**
dp vs. rp	*p* < 0.05	*p* < 0.0001	n.s.	n.s.	n.s.	n.s.	n.s.	n.s.
	n.s.	*p* <0.0001	n.s.	n.s.	*p* < 0.05	n.s.	n.s.	n.s.
dp vs. da	*p* < 0.01	*p* < 0.0001	*p* < 0.001	n.s.	n.s.	*p* < 0.0001	*p* < 0.0001	*p* < 0.0001
	*p* < 0.01	*p* <0.0001	n.s.	n.s.	n.s.	*p* < 0.0001	*p* < 0.0001	*p* < 0.0001
rp vs. da	n.s.	*p* < 0.0001	*p* < 0.0001	n.s.	n.s.	*p* < 0.0001	*p* < 0.01	*p* < 0.01
	n.s.	n.s.	n.s.	n.s.	n.s.	*p* < 0.0001	*p* < 0.01	*p* < 0.05

*Adjusted p-values (Tukey HSD) for comparisons of syllable duration (above) and vowel duration (below) between demonstrative pronouns (dp), relative pronouns (rp) and definite articles (da)*.

#### Prominence

**Figure 5:**
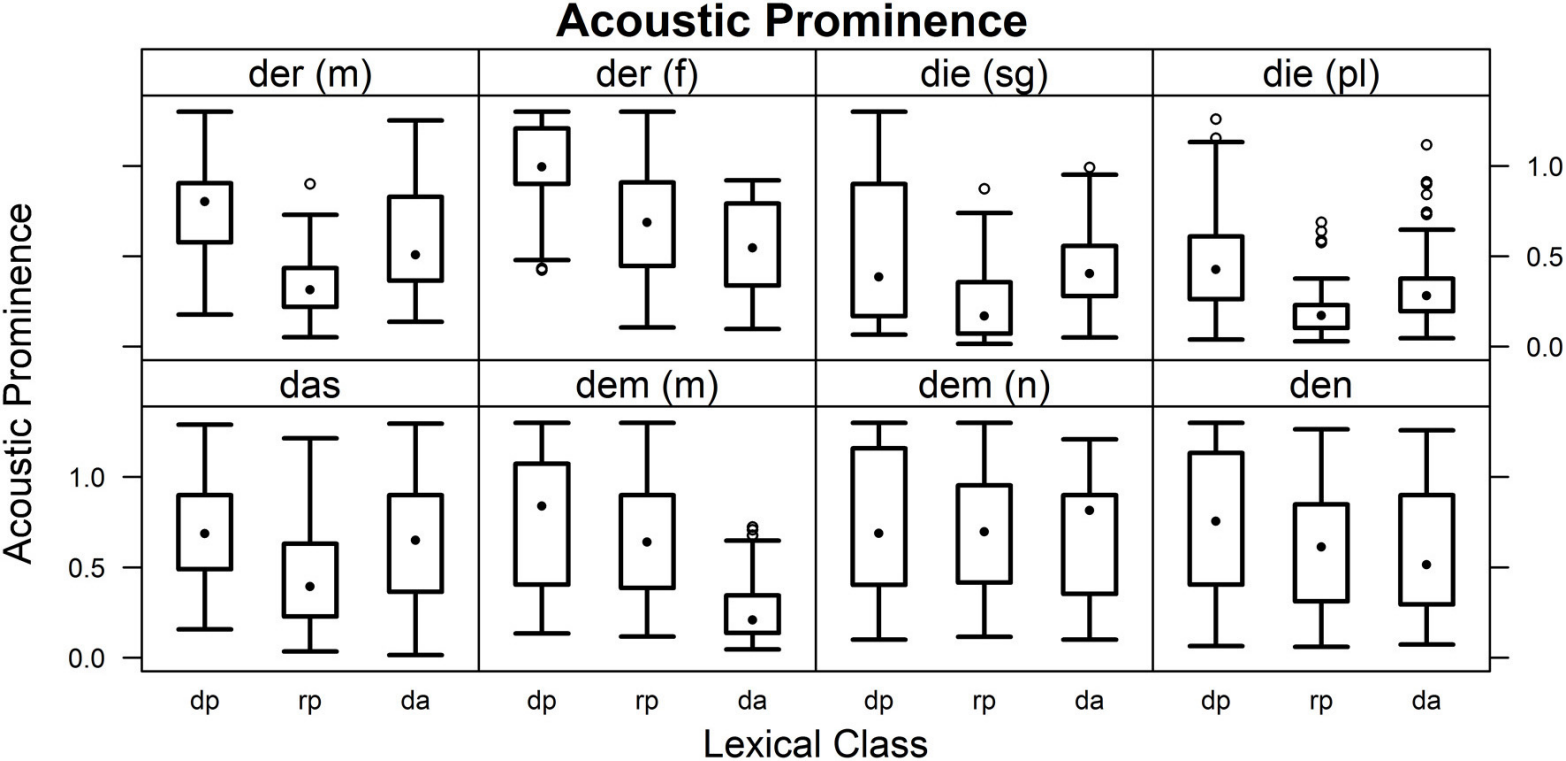
**Prominence values**. Estimates of acoustic prominence for the individual words in the roles of demonstrative pronoun (dp), relative pronoun (dp), and definite article (da).

Across items, prominences were higher for demonstrative pronouns than for relative pronouns and definite articles. Definite articles were minimally less prominent than relative pronouns. Figure 5 shows results by lexical class for the individual words. Combinations of word identity and lexical class were analyzed using Wilcoxon rank sum tests (corrected for 276 comparisons, see Table 8). No significant differences between lexical classes were found for neuter “dem” or “den.” For all other items except masculine “dem,” demonstrative pronouns tended to receive higher prominence values than relative pronouns. Demonstrative pronouns were more prominent than definite articles for masculine and feminine “der” and masculine “dem.” While definite articles tended to be more prominent than relative pronouns for masculine “der,” singular and plural “die,” and “das,” an opposite trend was visible for masculine “dem.”

**Table 8:** **Prominence statistics for differences in lexical class**.

**Lexical classes**	**der (masc)**	**der (fem)**	**die (sing)**	**die (plur)**	**das**	**dem (masc)**	**dem (neut)**	**den**
dp vs. rp	4922.5^****^	5067.0^****^	3935.5^***^	4934.5^****^	4840.5^****^	3089.0 (n.s.)	3286.5 (n.s.)	3061.5 (n.s.)
dp vs. da	3851.0^**^	5689.5^****^	3099.5 (n.s.)	4022.5 (n.s.)	3353.5 (n.s.)	5054.5^****^	3388.0 (n.s.)	3687.0 (n.s.)
da vs. rp	4937.5^****^	2135.5 (n.s.)	4739.0 ^****^	5144.4 ^****^	4566.0 ^**^	793.0 ^****^	2887.5 (n.s.)	2567.5 (n.s.)

W values with significance levels for comparisons between definite pronouns (dp), relative pronouns (rp) and definite articles (da), n.s.: p ≥ 0.05,

** p < 0.01,

*** p < 0.001,

**** *p < 0.0001*.

#### Spectral similarity

Similarity levels were computed for segmentally identical items belonging to the same lexical class and produced by the same speaker in different contexts. Across words, definite articles (mean value: 0.814) appeared to be minimally less consistent in their pronunciation than demonstrative or relative pronouns (mean values: 0.823, 0.823). The difference, however, was only significant in Wilcoxon rank sum tests (*W* > 739,000, *p* < 0.05, corrected for 3 comparisons) when similarities were calculated regardless of gender or class. No effects were found when word identity as well as segmental identity was controlled (corrected for 3 comparisons), or when lexical classes were compared separately for individual word identities (corrected for 276 comparisons). We also examined similarity levels between words belonging to different lexical classes (paired for speaker, word identity, and context). Here, we found a significant difference between similarity measures of relative and demonstrative pronouns on the one hand and relative pronouns and definite articles on the other (mean values: 0.861 vs. 0.850, *W* = 155,459.5, *p* < 0.05, corrected for 3 comparisons). In separate comparisons for individual word identities (corrected for 276 comparisons), this tendency was only confirmed for masculine “dem” (mean values: 0.893 vs. 0.831, *W* = 2651, *p* < 0.001).

#### Discussion

Definite articles were expected to have smaller duration values than segmentally identical relative or demonstrative pronouns due to effects of frequency and predictability. Not only are they much more common than the other lexical classes, the carrier sentences for the pronouns were specifically constructed to mirror the phonetic context of definite articles found in other sentences, probably increasing their artificiality and reducing the predictability of the target words. According to exemplar-theoretic approaches, definite articles might also be more strongly adapted to their surroundings, which would lead to lowered spectral similarity values across contexts. However, differences in pronunciation cannot always be explained by lemma frequency, and lexical classes may vary in the degree to which they can be emphasized. For instance, Jurafsky et al. (2000) found that although the English word “that” was most commonly used as a demonstrative pronoun, it tended to be longer in this function than when it was produced as a segmentally identical relative pronoun, complement, or determiner. In order to monitor for differences in emphasis, we also analyzed the target words' level of acoustic prominence in relation to their immediate context. In our investigation, we discovered significant differences between all three lexical classes in terms of syllable and vowel duration. Although these differences were not contradictory to lemma frequency effects, they did not mirror the fact that in German, frequency differences between the two types of pronouns are minimal compared to their difference to definite articles. The comparatively high duration of demonstrative pronouns was probably due to their semantic role, as it is their function to point out and emphasize the entity to which they refer. Results for acoustic prominence confirm that participants tended to emphasize demonstrative pronouns more strongly than relative pronouns or definite articles. Contrary to our expectations, we found only minimal effects and no consistent patterns in terms of spectral similarity within and between lexical classes.

A closer examination of the data revealed that the individual target words varied in the ways and extent to which they were affected by changes in lexical class. Duration differences were most stable in comparisons between demonstrative pronouns and definite articles. Relative pronouns often tended to be closer in duration to demonstrative pronouns than to definite articles. Plural “die” showed no duration effects whatsoever, and the only significant duration effect found for “das” was a slight difference in vowel duration between relative and demonstrative pronouns. Concerning acoustic prominence, it was striking that while any significant differences between demonstrative pronouns and definite articles was accompanied by significant effects of syllable and vowel duration, several words showed prominence differences between relative pronouns and the other two categories without any corresponding duration effects. Although relative pronouns were generally longer than definite articles, prominence levels tended to be lower, with only masculine “dem” showing a significant effect in the opposite direction. Only singular “die” showed contradictory duration and prominence results which were both significant. The conflicting prominence findings may have resulted from the difficulty in controlling the context of the target items. As relative pronouns are generally used to introduce relative clauses, the syllables preceding them tended to be clause-final and therefore subject to final lengthening. It is very likely that relative pronouns received particularly low prominence ratings by the tagger due to their relatively prominent preceding context. In the case of feminine “der,” masculine and neuter “dem,” and one sentence used for “den,” possible context lengthening was avoided by placing the relative pronouns in prepositional phrases. For these words, there was indeed no tendency for relative pronouns to be less prominent than definite articles, and prominence differences were supported by differences in syllable and vowel duration.

## Summary

This paper describes results from two experiments designed to disentangle various influences on syllable pronunciation in German. In the first experiment, we found clear differences due to word stress and sentence boundaries, while effects of sentence stress and word boundaries were smaller in size and less consistent across stimuli. In the second experiment, differences between segmentally identical demonstrative pronouns, relative pronouns, and definite articles were found that could be related to lemma frequency, semantic function, and sentence structure. In both experiments, duration was shown to be the most robust of the investigated cues for disambiguating word meanings. Measures of acoustic prominence added valuable information on how strongly syllables were emphasized, but also proved to be highly sensitive to differences in context. Finally, an examination of spectral similarity revealed that syllables in lexically stressed prefixes were less variable across contexts and closer in pronunciation to sentence-final realizations than unstressed prefixes. Separate investigations of individual target syllables often failed to reach significance in terms of acoustic prominence and spectral similarity, suggesting that other influences may also have been of importance. Especially prominence and similarity measures often failed to reach significance in these detailed analyses. A larger study covering a greater number of contexts and using a separate quasi-random order of sentences for each speaker, possibly followed by a perception study to confirm the results, might lead to more robust findings.

### Conflict of interest statement

The authors declare that the research was conducted in the absence of any commercial or financial relationships that could be construed as a potential conflict of interest.
