# Supplementary material for: Phonetic detail in German syllable pronunciation: influences of prosody and grammar
Source: Front Psychol. 2014 May 27;5:500. doi: 10.3389/fpsyg.2014.00500 (PMC4034494; doi:10.3389/fpsyg.2014.00500)
Supplement: Supplementary file 1 [file DataSheet1.PDF]

Supplementary Material

Phonetic Detail in German Syllable Pronunciation: Influences of Prosody and Grammar

Barbara Samlowski[1]*, Bernd Möbius[2], Petra Wagner[1]

[1]Work Group Phonetics/Phonology, Faculty of Linguistics and Literary Studies, Bielefeld University, Bielefeld, Germany
[2]Department of Computational Linguistics and Phonetics, Saarland University, Saarbrücken, Germany

*** Correspondence:** Barbara Samlowski, Work Group Phonetics/Phonology, Faculty of Linguistics and Literary Studies, Bielefeld University, Universitätsstraße 25, Bielefeld, 33615, Germany.
barbara.samlowski@uni-bielefeld.de

1. Stimuli for Experiment 1

Supplementary Table A. Sentences for "umfahren". "to drive over" ['ʔʊm.faː.ʁən] vs. "to drive around" [ʔʊm.'faː.ʁən]

**Category**	**Sentence + Translation**
w+s+	Die Kinder waren sehr traurig, als der Mann nicht bremsen konnte und ihre Katze **umfahren** musste.<br>*The children were very sad when the man wasn't able to brake and had to **run over** their cat.*
w+s-	Geländewagen können kleinere Sträucher überrollen, aber keine Bäume **umfahren**.<br>*SUVs can navigate over small bushes but cannot **drive over** trees.*
w-s+	Der Mann war in den Gegenverkehr geraten, als er die Katze **umfahren** wollte.<br>*The man swerved into the oncoming traffic as he tried to **drive around** the cat.*
w-s-	Das Boot muss sowohl die rote als auch die grüne Boje **umfahren**.<br>*The boat has to **drive around** the red as well as the green buoy.*
sb	Auf dem Weg nach Hause **fuhr** der Mann die Katze **um**.<br>*On his way home the man **ran over** the cat.*
mb	Die roten Bojen sollte man **umfahren**, wenn man sich nicht in Wasserpflanzen verheddern möchte.<br>*You should **drive around** the red buoys if you do not want to get tangled in seaweed.*
wb	Vor allem sollte man, **um Fahren** zu lernen, viel Geduld und Aufmerksamkeit mit sich bringen.<br>*What you need above all, **in order to** learn to **drive**, is a lot of patience and concentration.*

Supplementary Table B. Sentences for "umstellen". "to move around" ['ʔʊm.ʃtɛ.lən] vs. "to surround" [ʔʊm.'ʃtɛ.lən]

Category	Sentence + Translation
w+s+	Nach dem Umzug werden wir die Möbel vollkommen **umstellen**.<br>*After the move we are going to completely **re-arrange** the furniture.*
w+s-	Die Computer springen von selbst auf Sommerzeit um – ich muss nur noch die Uhren **umstellen**.<br>*The computers switch to daylight-saving time automatically – I only have to **re-set** the clocks.*
w-s+	Die Polizei konnte den Entführer vollkommen **umstellen**.<br>*The police managed to completely **surround** the kidnapper.*
w-s-	Die Polizei konnte das Haus, aber nicht den Garten **umstellen**.<br>*The police managed to **surround** the house but not the garden.*
sb	Nach dem Umzug **stellte** er seine Möbel vollkommen **um**.<br>*After the move, he completely **re-arranged** the furniture.*
mb	Die Polizei positionierte sich so, dass der Einbrecher sich **umstellt** fühlen musste.<br>*The police positioned themselves in a way which let the burglar feel himself **surrounded**.*
wb	Es handelt sich **um stellenweise** undichtes Material.<br>*It's **about partially** permeable material.*

Supplementary Table C. Sentences for "unterschlagen". "to fold in" ['ʔʊn.tɐ.ʃla:.gən] vs. "to embezzle" [ʔʊn.tɐ.'ʃla:.gən]

Category	Sentence + Translation
w+s+	Man muss die Butter **unterschlagen** und etwa drei Minuten verrühren, bis der Teig cremig wird.<br>*You have to **fold in** the butter and mix for three minutes until the dough is creamy.*
w+s-	Zunächst muss man Eier und Milch verrühren und anschließend den Zucker **unterschlagen**.<br>*First you have to mix the eggs with the milk and then **fold in** the sugar.*
w-s+	Sie hatte in dem Rezept aus Versehen Backpulver und Zucker **unterschlagen**.<br>*In the recipe, she accidentally **left out** the baking powder and the sugar.*
w-s-	Die Täterin hatte nicht nur zahlreiche Banken ausgeraubt, sondern auch Steuergelder **unterschlagen**.<br>*The culprit had not only robbed several banks but had also **embezzled** tax money.*
sb	Anschließend schlägt man Eier und Zucker **unter**.<br>*Then you **fold in** the eggs and the sugar.*
mb	Er kann sich die teuren Autos leisten, weil er regelmäßig Gelder **unterschlägt**.<br>*He can afford the expensive cars because he regularly **embezzles** money.*
wb	Schließlich gab er die Gelder **unter Schlägen** dem Räuber.<br>***Under blows**, he finally gave the money to the robber.*

Supplementary Table D. Sentences for "unterstellen". "to store / take shelter" ['ʔʊn.tɐ.ʃtɛ.lən] vs. "to insinuate" [ʔʊn.tɐ.'ʃtɛ.lən]

Category	Sentence + Translation
w+s+	Wir wollten uns **unterstellen**, weil es so stark regnet.<br>*We wanted to **take shelter** because it is raining so heavily.*
w+s-	Sie können nicht nur Fahrräder, sondern auch Autos bei uns **unterstellen**.<br>*You can **store** not only bicycles but also cars with us.*
w-s+	Der Kellner wollte uns **unterstellen**, dass wir nicht bezahlt hätten.<br>*The waiter wanted to **imply** that we hadn't paid.*
w-s-	Tierquälerei haben sie uns **unterstellt** – nur, weil unser Hund ihre Katze auf den Baum gejagt hat.<br>*They **accused** us of animal cruelty – just because our dog chased their cat up the tree.*
sb	Da es gerade stark regnete, **stellten** wir uns **unter**.<br>*Because it was raining heavily we **took shelter**.*
mb	Man kann **unterstellen**, dass Ratten von vielen als Ungeziefer gesehen werden.<br>*One can **assume** that rats are seen as vermin by many.*
wb	Man kann **unter Ställen** oft Mäuse und Ratten finden.<br>*One can often find rats and mice **under sheds**.*

Supplementary Table E. Sentences for "überlaufen". "to spill over" ['ʔyː.bɐ.laʊ.fən] vs. "to be crowded" [ʔyː.bɐ.'laʊ.fən]

Category	Sentence + Translation
w+s+	So sehr ich auch aufpasse – immer wird das Nudelwasser ein wenig **überlaufen**.<br>*As careful as I am – the pasta water will always **spill over** a bit.*
w+s-	Wenn du beim Eingießen acht gibst, wird nur wenig **überlaufen**.<br>*If you pour carefully, only a little will **spill over**.*
w-s+	Am Samstag Nachmittag ist die Stadt immer ein wenig **überlaufen**.<br>*On Saturday afternoon the town is always a little **crowded**.*
w-s-	Der Strand ist überall sehr voll, aber in diesem Abschnitt verhältnismäßig wenig **überlaufen**.<br>*The beech is very full everywhere, but this part is relatively less **crowded**.*
sb	Wenn es stark gießt, **läuft** die Regentonne immer ein wenig **über**.<br>*When it rains heavily, the rain barrel always **spills over** a little.*
mb	Joggen gehen sollte man dort eher abends, da der Park vorher ein wenig **überlaufen** ist.<br>*You should preferably go there in the evening to jog, since before then the park is a little **crowded**.*
wb	Wenn man joggen möchte, sollte man sich vorher ein wenig **über Laufen** als Sportart informieren.<br>*If you want to go jogging, you should learn a little **about running** as a sport beforehand.*

Supplementary Table F. Sentences for "überziehen". "to put on" ['ʔyː.bɐ.tsiː.ən] vs. "to overdraw" [ʔyː.bɐ.'tsiː.ən]

Category	Sentence + Translation
w+s+	Es ist kalt draußen – du solltest dir etwas **überziehen**.<br>*It is cold outside – you should **put** something **on**.*
w+s-	Nimm dein neues Jackett – bei so einem Fest sollte man sich nicht einfach irgendwas **überziehen**.<br>*Wear your new suit jacket – at a party like this you shouldn't **put on** just anything.*
w-s+	Es gibt drei Tage Kulanzzeit – Sie können also etwas **überziehen**.<br>*There is a grace period of three days – you can **overstep** (the deadline) a little.*
w-s-	Er würde sich nicht stark verschulden, sondern sein Konto nur etwas **überziehen**.<br>*He wouldn't go way into debt but just **overdraw** his account slightly.*
sb	Bevor er in die Kälte hinausging, **zog** er sich noch etwas **über**.<br>*Before he went out into the cold, he **put** something **on**.*
mb	Muss man sein Konto häufig **überziehen**, sollte man auf günstige Zinsen achten.<br>*If you need to frequently **overdraw** your account, you should look for favorable interest rates.*
wb	Er klagte häufig **über ziehende** Schmerzen in den Unterschenkeln.<br>*He frequently complained **about twinging** pains in his lower legs.*

Supplementary Table G. Sentences for "durchschauen". "to examine" ['dʊʁç.ʃaʊ.ən] vs. "to see through" ['dʊʁç.'ʃaʊ.ən]

Category	Sentence + Translation
w+s+	Der Lehrer hofft, dass er sich in den Ferien alle Arbeiten **durchschauen** kann.<br>*The teacher hopes that he can **look through** all the exams over the holidays.*
w+s-	Ich habe den Text fast ganz durchgesehen, ich muss mir nur noch die letzten Seiten **durchschauen**.<br>*I read almost the whole text; I only have to **look through** the last few pages.*
w-s+	Der Lehrer hofft, dass er die Plagiate in den Arbeiten **durchschauen** kann.<br>*The teacher hopes that he can **see through** plagiarism in the exams.*
w-s-	Der Junge ahnte, dass sein Vater ihn getäuscht hatte, konnte aber nicht den Grund für seine Lügen **durchschauen**.<br>*The boy suspected that his father had tricked him but couldn't **comprehend** the reason for his lies.*
sb	Der Lehrer **schaute** sich in den Ferien die Arbeiten **durch**.<br>*During the holidays, the teacher **looked through** the exams.*
mb	Der Wissenschaftler wollte den Kandidaten nur **durchschauen** und so seine Methoden herausfinden.<br>*The scientist only wanted to **figure out** the candidate and discover his methods.*
wb	Der Kandidat hat gewettet, dass er über 50 Teesorten nur **durch Schauen** unterscheiden kann.<br>*The candidate made a bet that he could distinguish 50 brands of tea merely **by looking** at them.*

Supplementary Table H. Sentences for "durchstreichen". "to cross out" ['dʊʁç.ʃtʁaɪ.çən] vs. "to wander through" [dʊʁç.'ʃtʁaɪ.çən]

Category	Sentence + Translation
w+s+	In dem Formular muss man alle leer gebliebenen Felder **durchstreichen**.<br>*On the form you should **cross out** any fields you left blank.*
w+s-	Bei der Korrektur sollte man nicht ganze Sätze, sondern nur die jeweiligen Fehler **durchstreichen**.<br>*During revision you should not **cross out** entire sentences but just the mistakes.*
w-s+	Auf ihrer Reise wollten sie Flüsse durchwaten und Felder **durchstreichen**.<br>*On their journey they wanted to wade through rivers and **wander through** fields.*
w-s-	Sie wollten keine Bergwanderung machen, sondern nur ein wenig die Felder **durchstreichen**.<br>*They didn't want to go hiking in the mountains, but just wanted to **wander through** the fields a little.*
sb	**Streichen** Sie in dem Antrag bitte alle nicht ausgefüllten Felder **durch**.<br>*Please **cross out** any fields you left blank in the application.*
mb	**Durchstreichen** Sie die zahlreichen Wälder oder genießen Sie die Aussicht vom Burgturm aus.<br>***Wander through** the many forests or enjoy the view from the castle tower.*
wb	**Durch Streichen** wird der Zaun vor Wind und Wetter geschützt.<br>***By painting,** you protect the fence from wind and weather.*

2. Stimuli for Experiment 2

Supplementary Table I. Sentences for "der (m)". [deːɐ̯] (masculine singular nominative)

Context	Lexical class	Sentence
1	Demonstr. Pron.	So, wie dieser Typ angezogen ist, hatte ich mir schon gedacht, dass **der** ein Feuerwehrmann ist.<br>*The way that guy was dressed, I already guessed that **that** was a fireman.*
	Rel. Pron.	Seitdem ihre Katze überfahren wurde, verzehrte sie ein Hass, **der** eines Tages ausbrechen würde.<br>*Since her cat was run over, she was consumed by a hatred **that** would some day explode.*
	Def. Art.	Die Polizei positionierte sich so, dass **der** Einbrecher sich umstellt fühlen musste.<br>*The police positioned themselves in a way which had to make **the** burglar feel himself surrounded.*
2	Demonstr. Pron.	Kann ich den 845er Bus nehmen? Früher fuhr **der** manchmal über Lüftelberg.<br>*Can I take bus number 845? **That** (bus) sometimes used to go via Lüftelberg.*

	Rel. Pron.	Der Fahrer des Wagens hinter der Müllabfuhr, **der** Maler von Beruf war, fand einfach keine Möglichkeit zum Überholen.<br>*The driver of the car behind the garbage truck,* ***who*** *was a painter, simply could not find a chance to pass it.*
	Def. Art.	Auf dem Weg nach Hause fuhr **der** Mann die Katze um.<br>*On the way home* ***the*** *man ran over the cat.*
3	Demonstr. Pron.	Der Flug hat Verspätung – es dauert noch eine ganze Weile, bis **der** Thailand erreicht.<br>*The flight is delayed – it will take some time before* ***that*** *(flight) reaches Thailand.*
	Rel. Pron.	Die Schlange hat einen Biss, **der** teilweise lähmend wirkt.<br>*The snake has a bite* ***which*** *is partially paralyzing.*
	Def. Art.	Man muss die Butter unterschlagen und etwa drei Minuten verrühren, bis **der** Teig cremig wird.<br>*You have to fold in the butter and mix for three minutes until* ***the*** *batter is creamy.*

Supplementary Table J. Sentences for "der (f)". [deːɐ] (feminine singular dative)

Context	Lexical class	Sentence
1	Demonstr. Pron.	Die Eisdiele ist sehr teuer - bei **der** kostet eine Kugel 1,20 Euro.<br>*The ice cream parlor is very expensive – at* ***that*** *(place) one scoop costs 1.20 euros.*
	Rel. Pron.	Vor der Fahrt gibt es eine Routineuntersuchung, bei **der** kontrolliert wird, ob alle Gurte fest sitzen<br>*Before the ride there is a routine inspection, during* ***which*** *they check to see whether all the belts are securely fastened.*
	Def. Art.	Bei **der** Korrektur sollte man nicht ganze Sätze, sondern nur die jeweiligen Fehler durchstreichen.<br>*During (****the****) revision you should not cross out entire sentences, but just the mistakes.*
2	Demonstr. Pron.	Für ein kräftiges Rosa muss man eine Brühe aus roter Beete kochen – in **der** färbt man dann die Eier.<br>*For bright pink you need to make a dye from of red beets – in* ***that*** *(dye) you then color the eggs.*
	Rel. Pron.	Sie mochte diese Geschichte, in **der** ferne Länder und fremdartige Kulturen beschrieben wurden.<br>*She liked this story, in* ***which*** *far-away countries and strange cultures were described.*
	Def. Art.	In **der** Ferne ist ein Kahn zu sehen – auf dem weht eine amerikanische Fahne.<br>*In* ***the*** *distance you can see a boat – on that (boat) an American flag is waving.*
3	Demonstr. Pron.	Die Kutsche darf die Kreuzung nicht als erste überqueren – vor **der** fahren zunächst der Käfer und dann der LKW.<br>*The horse-drawn carriage may not cross the intersection first – before* ***that*** *(vehicle), the VW and then the truck have right of way.*

	Rel. Pron.	Es gibt eine Hütte neben dem Haus, vor **der** Fahrräder abgestellt werden können.<br>*There is a shed alongside the house, in front of* ***which*** *bicycles can be stored.*
	Def. Art.	Vor **der** Fahrt gibt es eine Routineuntersuchung, bei der kontrolliert wird, ob alle Gurte fest sitzen.<br>*Before* ***the*** *ride there is a routine inspection, during which they check to see whether all the belts are securely fastened.*

Supplementary Table K. Sentences for "die (sg)". [di:] (feminine singular nominative/accusative)

Context	Lexical class	Sentence
1	Demonstr. Pron.	Die Frau kam ihm vertraut vor, aber er hatte keine Ahnung, woher er **die** kannte.<br>*The woman seemed familiar to him, but he had no idea from where he knew* ***that*** *(woman).*
	Rel. Pron.	Das Mädchen mochte die Geschichte sehr, **die** Kaninchen, Ponys und Drachen enthielt.<br>*The girl very much liked the story,* ***which*** *contained rabbits, ponies, and dragons.*
	Def. Art.	Der Mann war in den Gegenverkehr geraten, als er **die** Katze umfahren wollte.<br>*The man swerved into the oncoming traffic as he tried to drive around* ***the*** *cat.*
2	Demonstr. Pron.	Das Auto hat die Ampel überfahren, obwohl **die** rot war.<br>*The car went through the traffic light even though* ***that*** *(light) was red.*
	Rel. Pron.	Da war eine Schnecke im Kohl, **die** rosa und schleimig zwischen den Blättern hervorlugte.<br>*There was a snail in the cabbage,* ***which*** *peeped out pink and slimy from between the leaves.*
	Def. Art.	Das Boot muss sowohl **die** rote als auch die grüne Boje umfahren.<br>*The boat has to drive around* ***the*** *red as well as the green buoy.*
3	Demonstr. Pron.	Diese Suppe ist sehr vielseitig – man kann **die** kalt oder warm genießen.<br>*The soup is very versatile – you can enjoy* ***that*** *(soup) cold or warm.*
	Rel. Pron.	Eine neue Ära begann, **die** Kathedralenbau überall in Europa revolutionieren sollte.<br>*A new era began* ***which*** *would revolutionize the building of cathedrals everywhere in Europe.*
	Def. Art.	Auf dem Weg nach Hause fuhr der Mann **die** Katze um.<br>*On the way home the man ran over* ***the*** *cat.*

Supplementary Table L. Sentences for "die (pl)". [di:] (masculine/feminine/neuter plural nominative/accusative)

Context	Lexical class	Sentence
1	Demonstr. Pron.	Ich finde die Brötchen nicht – haben wir **die** möglicherweise vergessen?<br>*I cannot find the rolls – might we have forgotten **those**?*
	Rel. Pron.	Da sind ein paar Noten auf dem Klavier, **die** möglicherweise dir gehören.<br>*There are some notes on the piano **which** might belong to you.*
	Def. Art.	Nach dem Umzug werden wir **die** Möbel vollkommen umstellen.<br>*After the move we are going to completely re-arrange **the** furniture.*
2	Demonstr. Pron.	Aus den Äpfeln würde er Kompott machen – zwar wollte der Koch **die** ursprünglich für einen Kuchen verwenden, doch das Mehl war ausgegangen.<br>*He would make stew out of the apples – the cook originally wanted to use **those** for a cake, but there was no flour left.*
	Rel. Pron.	Er besaß die Fische immer noch, **die** ursprünglich seiner Schwester gehört hatten.<br>*He still owned the fishes **which** had originally belonged to his sister.*
	Def. Art.	Die Computer springen von selbst auf Sommerzeit um – ich muss nur noch **die** Uhren umstellen.<br>*The computers switch to daylight-saving time automatically – I only have to re-set **the** clocks.*
3	Demonstr. Pron.	Eine exakte Aussprache ist wichtig für Sänger von Opernarien – **die** artikulieren jede Silbe überdeutlich.<br>*An exact pronunciation is important for singers of operatic arias – **those** (singers) exaggerate their articulation of every syllable.*
	Rel. Pron.	Panik wurde ausgelöst durch Bären, **die** arglos in die Dörfer eindrangen, um Mülltonnen zu durchstöbern.<br>*Panic was caused by bears **which** naively entered the villages to search through garbage cans.*
	Def. Art.	Der Lehrer schaute sich in den Ferien **die** Arbeiten durch.<br>*During the holidays, the teacher looked through **the** exams.*

Supplementary Table M. Sentences for "das". [das] (neuter singular nominative/accusative)

Context	Lexical class	Sentence
1	Demonstr. Pron.	Es handelte sich um ein Piratenschiff – er erkannte **das** hauptsächlich an der schwarzen Flagge mit dem Totenkopf.<br>*It was a pirate ship – he recognized **that** mainly from the black flag with the skull and crossbones.*
	Rel. Pron.	Sie wusste, dass sie dem Tier nicht zu nahe kommen sollte, **das** hauptsächlich wegen seines Stachels gefürchtet war.<br>*She knew that she should not come too close to the animal **which** was mainly feared because of its sting.*

	Def. Art.	Die Polizei konnte **das** Haus, aber nicht den Garten umstellen.<br>*The police managed to surround* ***the*** *house but not the garden.*
2	Demonstr. Pron.	Egal, wie viele Luftballons der Junge hat, kann er damit nicht fliegen – er wird **das** nur aus Erfahrung lernen können.<br>*Regardless of how many balloons the boy has, he will not be able to fly with them – he will learn* ***that*** *only through experience.*
	Rel. Pron.	Das ist die Fliege, die um das Flusspferd schwirrt, **das** nur seine Ruhe haben möchte.<br>*That is the fly which is buzzing around the hippopotamus* ***that*** *only wants to be left in peace.*
	Def. Art.	So sehr ich auch aufpasse – immer wird **das** Nudelwasser ein wenig überlaufen.<br>*As careful as I am –* ***the*** *pasta water will always spill over a bit.*
3	Demonstr. Pron.	Der Junge lässt ein großes Blatt von der Brücke fallen, um **das** flussabwärts treiben zu sehen.<br>*The boy drops a large leaf from the bridge in order to see* ***that*** *(leaf) float downstream.*
	Rel. Pron.	In dem Haus geht ein Gespenst um, **das** fluchtartig davonhuscht, sobald sich ein Mensch in der Nähe blicken lässt.<br>*The house is haunted by a ghost* ***that*** *hastily disappears as soon as a human being approaches.*
	Def. Art.	Das ist die Fliege, die um **das** Flusspferd schwirrt, das nur seine Ruhe haben möchte.<br>*That is the fly which is buzzing around* ***the*** *hippopotamus that only wants to be left in peace.*

Supplementary Table N. Sentences for "dem (m)". [deːm] (masculine singular dative)

**Context**	**Lexical class**	**Sentence**
1	Demonstr. Pron.	Sie hatte ihren Fahrradschlüssel verlegt und wollte sich hier nach **dem** umsehen.<br>*She lost her bicycle key and wanted to look for* ***that*** *(key) here.*
	Rel. Pron.	Der Junge beschrieb der Polizei seinen Entführer, nach **dem** umgehend eine Großfahndung eingeleitet wurde.<br>*The boy gave the police a description of the kidnapper, after* ***whom*** *a man hunt was immediately organized.*
	Def. Art.	Nach **dem** Umzug werden wir die Möbel vollkommen umstellen.<br>*After* ***the*** *move we are going to completely re-arrange the furniture.*
2	Demonstr. Pron.	In der Ferne ist ein Kahn zu sehen – auf **dem** weht eine amerikanische Fahne.<br>*In the distance you can see a boat – on* ***that*** *(boat) an American flag is waving.*
	Rel. Pron.	Er lief den Strand entlang, auf **dem** wegen des anhaltenden Regens kaum jemand zu sehen war.<br>*He ran along the beach, on* ***which****, due to the continuing rain, hardly anybody could be seen.*

	Def. Art.	Auf **dem** Weg nach Hause fuhr der Mann die Katze um.<br>*On **the** way home the man ran over the cat.*
3	Demonstr. Pron.	Er hatte sich für seine Forschungsergebnisse einen Safe gekauft – niemand ahnte, dass er in **dem** andere Dinge aufbewahrte als Bargeld.<br>*He had bought a safe for his research results – no one suspected that he kept other things in **that** (safe) than cash.*
	Rel. Pron.	Es gab einen gesonderten Karton für Pralinen und einen kleinen Korb, in **dem** andere Süßigkeiten aufbewahrt wurden.<br>*There was a separate box for chocolates and a small basket in **which** other sweets were kept.*
	Def. Art.	Streichen Sie in **dem** Antrag bitte alle nicht ausgefüllten Felder durch.<br>*Please cross out any fields you left blank in **the** application.*

Supplementary Table O. Sentences for "dem (n)". [deːm] (neuter singular dative)

**Context**	**Lexical class**	**Sentence**
1	Demonstr. Pron.	Sie hatte sich ein gelbes Notizbuch gekauft und wollte in **dem** Rezepte aufschreiben.<br>*She had bought a yellow notebook and wanted to write down recipes in **that** (notebook).*
	Rel. Pron.	Die Apotheke hat ein abgetrenntes Zimmer, in **dem** rezeptpflichtige Arzneimittel aufbewahrt werden.<br>*The pharmacy has a separate room, in **which** prescription drugs are stored.*
	Def. Art.	Sie hatte in **dem** Rezept aus Versehen Backpulver und Zucker unterschlagen.<br>*In **the** recipe, she had inadvertently left out the baking powder and the sugar.*
2	Demonstr. Pron.	Der Mann lief aus der Bank zu seinem Auto und fuhr in **dem** fort, ohne den zweiten Wagen zu bemerken, der ihm unauffällig folgte.<br>*The man ran from the bank to his car and drove away in **that** (car) without noticing the second car which followed him inconspicuously.*
	Rel. Pron.	Hier ist das Delphinbecken, in **dem** Forschungen zum Verhalten von Meeressäugern durchgeführt werden.<br>*Here is the dolphin tank, in **which** research is conducted on the behavior of marine mammals.*
	Def. Art.	In **dem** Formular muss man alle leer gebliebenen Felder durchstreichen.<br>*On **the** form you should cross out any fields you left blank.*
3	Demonstr. Pron.	Von der Verpflegung her ist unser Kaninchen sehr unkompliziert – wir geben **dem** hauptsächlich Gras und Heu.<br>*Our rabbit is very uncomplicated concerning its diet – we mainly give **that** (rabbit) grass and hay.*

	Rel. Pron.	Auf dem Grundstück, neben **dem** haushoch ein Baum steht, ist ein Spielplatz.<br>*On the property, alongside **which** there is a tree as high as a house, there is a playground.*
	Def. Art.	Es gibt eine Hütte neben **dem** Haus, vor der Fahrräder abgestellt werden können.<br>*There is a shed alongside **the** house, in front of which bicycles can be stored.*

Supplementary Table P. Sentences for "den". [deːn] (masculine singular accusative)

Context	Lexical class	Sentence
1	Demonstr. Pron.	Der Hund hatte den Hasen schon so oft weglaufen sehen - er wollte **den** endlich einmal fangen.<br>*The dog had seen the hare run away so often – he finally wanted to catch **that** (hare).*
	Rel. Pron.	Es war deutlich, dass der Fuchs den See beobachtete, **den** Enten als ihre Heimat gewählt hatten.<br>*It was clear that the fox was watching the lake **which** ducks had chosen as their home.*
	Def. Art.	Die Polizei konnte **den** Entführer vollkommen umstellen.<br>*The police managed to completely surround **the** kidnapper.*
2	Demonstr. Pron.	Das Auto hat einen großen Kofferraum - in **den** geht eine ganze Menge hinein.<br>*The car has a large luggage compartment – in **that** (compartment) you can fit a great deal.*
	Rel. Pron.	Zum Burgturm fährt in regelmäßigen Abständen ein kleiner Bus, in **den** Gehfaule steigen können.<br>*At regular intervals, a small bus drives to the castle tower, into **which** those who are tired of walking can climb.*
	Def. Art.	Der Mann war in **den** Gegenverkehr geraten, als er die Katze umfahren wollte.<br>*The man swerved into **the** oncoming traffic as he tried to drive around the cat.*
3	Demonstr. Pron.	Der neue Gasherd war ihr immer noch suspekt und sie versäumte nicht, **den** grundsätzlich auszumachen, sobald sie die Küche verließ.<br>*She was still wary of the new gas oven and did not fail to turn **that** (oven) off as a matter of principle whenever as she left the kitchen.*
	Rel. Pron.	Er mochte den Lärm nicht, **den** grunzende Ferkel verursachen.<br>*He did not like the noise **which** grunting piglets made.*
	Def. Art.	Der Junge ahnte, dass sein Vater ihn getäuscht hatte, konnte aber nicht **den** Grund für seine Lügen durchschauen.<br>*The boy suspected that his father had tricked him but couldn't comprehend **the** reason for his lies.*

3. Example illustrations (Experiment 1 – "unterstellen")

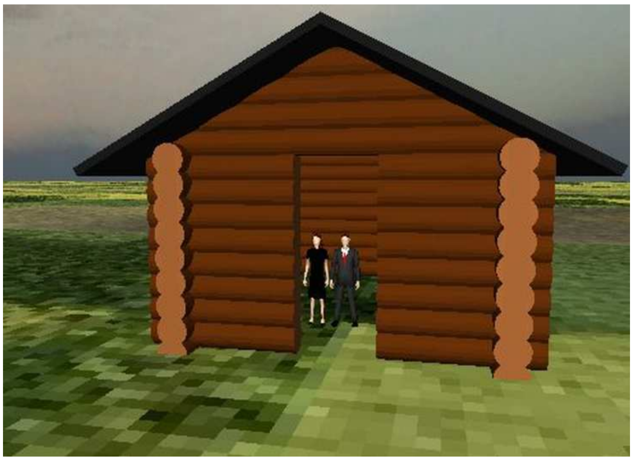

Figure A. Illustration for Sentence 1 (w+s+). "Wir wollten uns unterstellen, weil es so stark regnet." (We wanted to take shelter because it is raining so heavily.)

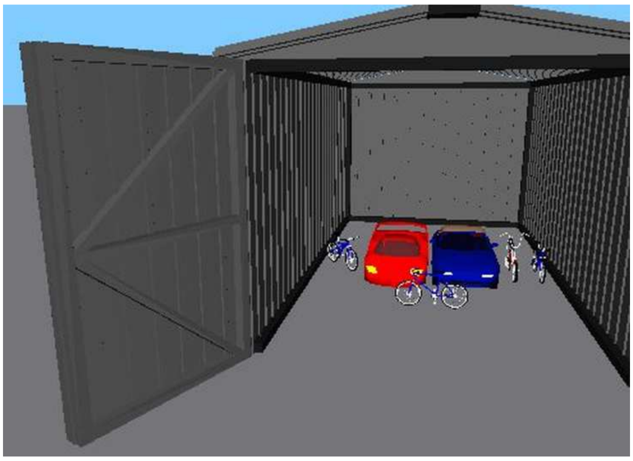

Figure B. Illustration for Sentence 2 (w+s-). "Sie können nicht nur Fahrräder, sondern auch Autos bei uns unterstellen." (You can store not only bicycles but also cars with us.)

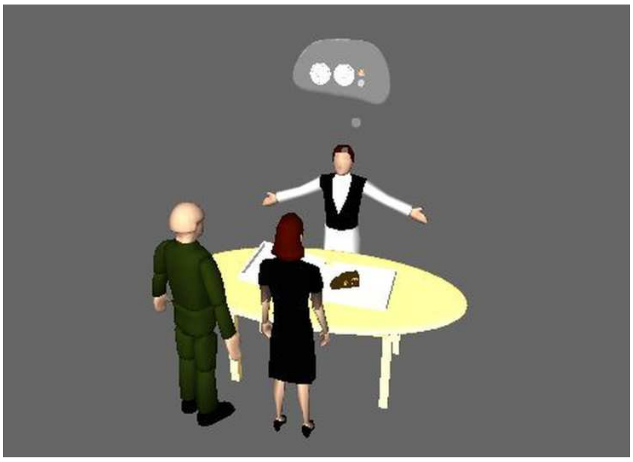

Figure C. Illustration for Sentence 3 (w-s+). "Der Kellner wollte uns unterstellen, dass wir nicht bezahlt hätten." (The waiter wanted to imply that we hadn't paid.)

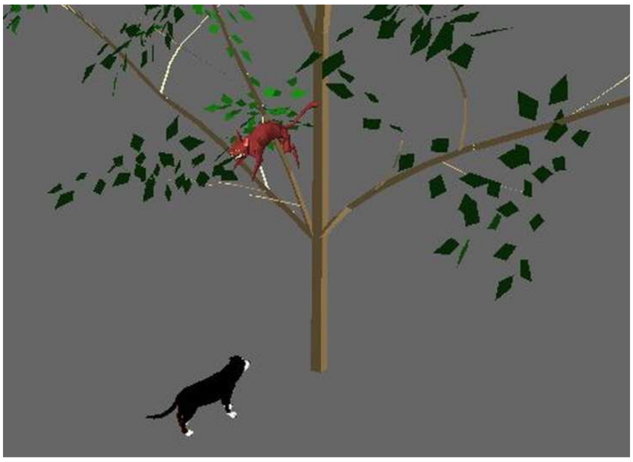

Figure D. Illustration for Sentence 4 (w-s-). "Tierquälerei haben sie uns unterstellt – nur weil unser Hund ihre Katze auf den Baum gejagt hat." (They accused us of animal cruelty – only because our dog had chased their cat up the tree.)

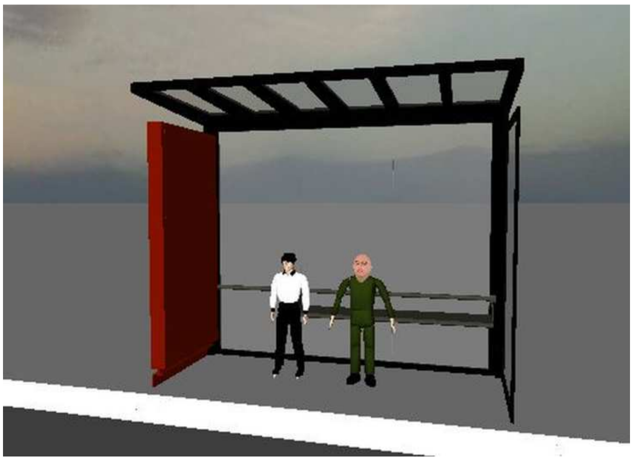

Figure E. Illustration for Sentence 5 (sb). "Da es gerade stark regnete, stellten wir uns unter." (Because it was raining heavily we took shelter.)

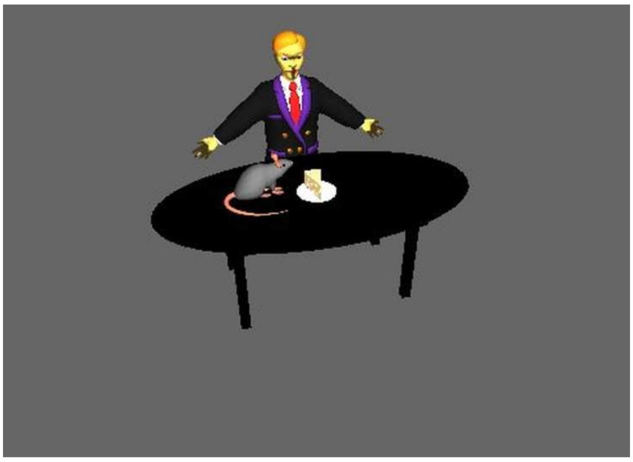

Figure F. Illustration for Sentence 6 (mb). "Man kann unterstellen, dass Ratten von vielen als Ungeziefer gesehen werden." (One can assume that rats are seen as vermin by many.)

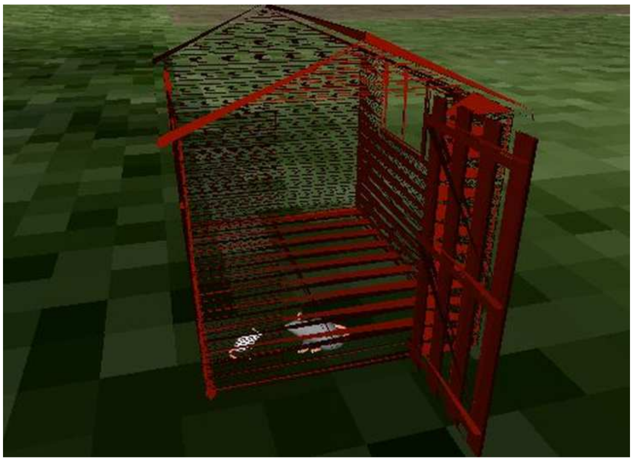

Figure G. Illustration for Sentence 7 (wb). "Man kann unter Ställen oft Mäuse und Ratten finden." (One can often find mice and rats under sheds.)
